# Interpersonal relations of pregnant women post-HIV diagnosis in Thembisile Hani, South Africa

**DOI:** 10.4102/sajhivmed.v25i1.1634

**Published:** 2024-12-20

**Authors:** Andile G. Mokoena-de Beer, Sister V. Mahlangu, Eugene M. Makhavhu

**Affiliations:** 1 Department of Nursing Science, School of Health Care Sciences, Sefako Makgatho Health Sciences University, Pretoria, South Africa

**Keywords:** HIV, interpersonal relationships, pregnancy, psychosocial support services, stigmatisation, women

## Abstract

**Background:**

HIV is a major public health issue in South Africa, with around 7.7 million people living with the virus by 2023, including 4.9 million women. In 2022, 257 171 pregnant women received antiretroviral therapy to prevent mother-to-child transmission.

**Objectives:**

To explore and describe the interpersonal relationships of pregnant women following HIV diagnosis in the Thembisile Hani Municipality, South Africa.

**Method:**

An exploratory descriptive qualitative design was used. Twenty (20) women aged 18–35 years, who were diagnosed with HIV during pregnancy, were purposively selected from a local clinic in Thembisile Hani Municipality. Data were collected through unstructured face-to-face interviews and analysed using thematic analysis.

**Results:**

Two themes emerged from the analysis; namely: (1) altered relationships with loved ones and (2) the role of psychosocial support to improve interpersonal relationships. These results indicate that being diagnosed with HIV during pregnancy has a negative impact on the interpersonal relationships of women.

**Conclusion:**

HIV diagnosis during pregnancy affects relationships, necessitating psychosocial support services such as counselling and support groups to improve well-being and relationship quality in pregnant women.

**What this study adds:** The findings of this study provide insights on the need to consider ongoing support strategies during antenatal care for pregnant women diagnosed with HIV, including involving their intimate partners, families, friends, and colleagues.

## Introduction

HIV is a public health concern in South Africa, with around 7.7 million people living with the virus by 2023, including 4.9 million women..^1,2^ In 2022, 257 171 pregnant women received antiretroviral therapy (ART) to prevent mother-to-child transmission.^2^ The prevalence of HIV among pregnant women in South Africa is much higher than in other African countries such as Nigeria, which had 32 276, Zimbabwe, with 43 856, and Lesotho, 6531, by 2022.^2,3^

The World Health Organization (WHO)^4^ recommends that at least one integrated antenatal care (ANC) visit be linked to HIV testing. Furthermore, the National Integrated Maternal and Perinatal Care Guidelines for South Africa^5^ mandate that HIV testing should be offered to all pregnant women with unknown or HIV-negative status. Thus, HIV testing is amplified in the WHO Guidelines on HIV prevention, testing, treatment, service delivery, and monitoring.^4^ The prevention of vertical transmission of HIV, syphilis, and other infections is the third goal of the guidelines for vertical transmission of communicable infections in South Africa. As a result, one of the pillars of the guidelines is the prevention of transmission from a woman living with a transmissible disease, such as HIV, to her infant.^6^ In the absence of any form of intervention, there is a 15% – 30% risk that an HIV-infected mother will transmit infection to her infant, largely in the intrapartum period.^7^ Due to this, early prenatal booking is encouraged to facilitate HIV testing for all expectant mothers who are unsure of their status and those who tested negative prior to pregnancy.

HIV poses potential adverse outcomes for both the mother and the unborn child. Some of the known adverse outcomes include mother-to child transmission (MTCT), with a high risk if the mother is not initiated on ART, preterm birth, low birth weight, increased infant mortality, and developmental delays.^8,9,10^ To reduce the risk of MTCT in South Africa, HIV testing has become mandatory.

Women living with HIV are more vulnerable to stress because of the difficulties in navigating pregnancy together with HIV diagnosis during pregnancy.^11^ Each additional clinic visit or laboratory test can increase emotional distress including denial, fear, anxiety, or concerns about external disclosure.^12^ Incorporating testing into routine prenatal care and providing culturally sensitive services may ensure more successful HIV testing for pregnant women, and at the same time take steps to prevent HIV transmission to the partner who is negative.^9^ Unsanctioned disclosure of a woman’s HIV status during testing or early provision of support can undermine her relationship with her partner or her safety at home, since most women do not disclose because they are unsure whether the pregnancy will be desired or not.^13^ An unwanted test result, where professional support is not readily available, can add to the emotional turmoil of pregnancy.^12^ Focusing solely on caregiving, without also addressing the pregnant woman’s need for specific psychological and social support, may compound her distress, and strain her ability to cope with her diagnosis.

Although it may be beneficial for a pregnant woman to test for HIV during pregnancy, the implications on interpersonal relationships are such that abandonment or divorce may be inevitable.^14^ While healthcare facilities may do their best to ensure that women are taken care of by initiating early ART, reactions from loved ones and the impact on mental health cannot be controlled. A qualitative study conducted in Cape Town found that women who disclosed their HIV status experienced complexities within their relationships, such as conflict and abuse, which were directly related to lack of partner support.^15^ Stigma and discrimination are still very much associated with the diagnosis of HIV, from healthcare providers, close relatives, and communities, and has a debilitating effect on the mental health of those diagnosed with HIV.^16,17,18^ The diagnosis of HIV is inevitably linked to psychosocial impact, including feelings of shame that impact on their relationships and subsequently restrict the extent of support received from loved ones.^19,20^

### Purpose of the study

This study explored the interpersonal relationships of pregnant women following HIV diagnosis in the Thembisile Hani Municipality, South Africa.

## Research methods and design

### Research design

An exploratory descriptive qualitative design was utilised to obtain an in-depth understanding of the interpersonal relationships of pregnant women following HIV diagnosis.

### Study setting

The study was conducted at a local clinic in the Thembisile Hani Municipality within the Nkangala District in Mpumalanga province, South Africa. The municipality is a semiurban local municipality consisting of 57 villages within five established townships, with a total population of 431 248, where 52.2% are women as recorded by the census 2022.^21^ The unemployment rate as of 2022 is 34.4%, while 75.2% of the population has some form of educational institution attendance. By 2022, Mpumalanga province recorded the highest rates of HIV prevalence in South Africa, with approximately 17.4%, translating to an estimated 890 000 people living with HIV in the province.^22^ Women in the province have a 1.6-fold (31.9%) higher HIV prevalence than men, with 19.9% among adults aged 25–49 years. ART coverage was substantially lower among women aged 15–24 years (58.6%) than among those aged 25–49 years (85.2%).^22^ Thembisile Hani Municipality has two clinics in the neighbourhood, along with a community health centre; both settings offer ANC Prevention of Mother-to-Child Transmission of HIV, chronic disease, minor illness programmes, immunisation extensions, and integrated management of childhood illnesses to the community. The Thembisile Hani Municipality clinics and the community health centre serve 29 279 women living with HIV, including pregnant women. The setting was selected because of the large number of HIV-positive women receiving care at this clinic.

### Population and sampling

The study enrolled 20 women who had been diagnosed with HIV during the current pregnancy or any of their previous pregnancies. They were purposively selected following being diagnosed with HIV during pregnancy. The sampling criteria required the women to have received an HIV diagnosis during pregnancy, have received treatment at the local clinic in the Thembisile Hani Municipality, and fall within the age range of 18–55 years. The sample size was informed by saturation point, which is the time during data collection where the phenomenon of interest had been fully described and understood and there was no new information that added to the understanding of the phenomenon.^23,24^

### Recruitment process

After obtaining ethical approval, the researchers obtained permission from the Mpumalanga Department of Health and the clinic manager for access to the Tier.net system to identify the women who were diagnosed with HIV during pregnancy. Tier.net is an electronic patient management system that is used for monitoring and evaluation of HIV care and treatment programmes in government health facilities throughout South Africa. Furthermore, the system was used to extract contact details of the women as allowed by the department and clinic manager; then, individuals were contacted to explain the purpose of the study. Although they were initially sceptical, they were willing to participate in the study. Additionally, those who were interested agreed to follow up individual appointments for the purpose of obtaining informed consent. As such, the researchers only included women who were willing to participate in the study. However, each participant had the same opportunity to participate.

### Data collection

Unstructured face-to-face interviews were conducted by the second author with each woman who agreed to participate in this study. All interviews were in English, at a time convenient for the participants, and lasted 20 min – 35 min. The interviews were recorded and stored using an audio recorder with the participants’ consent. A broad opening question allowed the women to elaborate on their interpersonal relationships following diagnosis with HIV during pregnancy.^25,26^ The question was, ‘How did the diagnosis of HIV affect your relationship with those around you?’ Follow-up questions were used as probes to clarify unclear statements and for in-depth exploration, allowing the women to elaborate further. Persistent observation throughout data collection, using field notes and observations, helped establish the credibility of the findings. Additionally, to extend interaction with participants, the researcher conducted interviews until the point of saturation.^25^ The interviews were transcribed and recorded verbatim to guarantee dependability. Debriefing was provided in instances where participants felt emotionally distressed.

### Data analysis

The data were transformed into written transcripts. The researcher identified themes as told by the women using thematic analysis. Thematic analysis in this study involved the following steps: reading the transcripts several times, developing codes, sorting the codes, developing a coding system by breaking down themes into sub-themes, labelling the themes and, finally, presenting the final themes.^27^ An independent coder analysed the data independently from the researchers. Subsequently, a consensus meeting was organised to reach a consensus on the identified themes. Literature control was used to confirm findings against previously published research to ensure their confirmability.^25^

### Ethical considerations

The study was approved by the Sefako Makgatho Health Sciences University Ethics Committee (reference no.: SMUREC/H/341/2023:PG). Furthermore, permission to conduct the study was sought from the Department of Health Mpumalanga, who gave approval to access the facility where the data were collected. The operational manager also gave permission for access to the Tier.net system used to keep records of those diagnosed with HIV, including pregnant women. The women voluntarily consented to participate in the study. No identifying data were revealed in relation to the data they provided.

## Results

### Characteristics of the participants

Twenty women who were diagnosed with HIV during pregnancy and aged between 20 years and 47 years, with a mean age of 33 years, participated in the study. Gravidity at the time of data collection ranged between one and seven pregnancies, and was self-reported. Seven (35%) lived with their parents, six (30%) lived with their life partners, five (25%) lived with their siblings, and two (10%) lived with only their children (Table 1).

**TABLE 1:** Characteristics of the study participants (*N* = 20 women diagnosed with HIV during pregnancy).

Participant no.	Age (years)	Gravidity	Gravidity at the time of diagnosis	Residing with at the time of diagnosis	Disclosed HIV status to
Participant 1	23	1	1	Mother	Partner
Participant 2	33	6	3	Life partner	Life partner
Participant 3	28	3	3	Sister and nephew	Sister
Participant 4	33	4	3	Sister and children	Family
Participant 5	36	6	4	Life partner and children	Previous fiancé
Participant 6	21	1	1	Sister	Sister and mother
Participant 7	28	4	2	Siblings	Aunt
Participant 8	34	4	1	Life partner and child	Life partner
Participant 9	32	2	2	Parents	Life partner
Participant 10	42	3	3	Siblings	Siblings
Participant 11	28	3	1	Mother	Mother
Participant 12	23	1	1	Mother	Mother
Participant 13	47	7	5	Children	No one
Participant 14	35	2	2	Life partner	Life partner
Participant 15	32	2	2	Mother	Mother and life partner
Participant 16	36	1	1	Life partner	Life partner
Participant 17	20	1	1	Mother	Mother
Participant 18	38	2	1	Parents	Parents
Participant 19	35	2	2	Life partner	Aunt
Participant 20	43	2	1	Child	Mother

### Key themes

Two overarching themes emerged from the data analysis, reflecting: (1) altered relationships with significant others, and (2) the role of psychosocial support to improve interpersonal relationships. These themes and sub-themes are presented in Table 2.

**TABLE 2:** Overview of the themes and sub-themes.

Themes		Sub-themes
1.	Altered relationships with significant others	1.1 Attitudes of life partners after the disclosure of HIV 1.2 The lack of support from family members 1.3 Stigmatising behaviours from friends and colleagues
2.	The role of psychosocial support to improve interpersonal relationships	2.1 The value of sound interpersonal relationships with significant others 2.2 Counselling services 2.3 Support groups

### Theme 1: Altered relationships with significant others

This theme describes the participants’ experience of altered relationships following diagnosis with HIV during pregnancy. The diagnosis of HIV during pregnancy significantly affected women’s relationships with their life partners, family, friends, and colleagues. The sub-themes include attitudes from their life partners, the lack of support from family members, and stigmatising behaviours from friends and colleagues.

#### Sub-theme 1.1: Attitudes of life partners after the disclosure of HIV

The women described that the disclosure of their HIV status to their life partners resulted in strained relationships. They began to experience negative attitudes from their life partners, which negatively impacted the intimate relationships of the women. Participants said:

‘Sometimes he comes to the house and insult me for no reason so that does not sit well with me.’ (Participant 10)

‘Initially it was rocky but now it is fine.’ (Participant 18)

‘Our relationship was a mess.’ (Participant 11)

‘It was no longer nice because even my libido decreased towards him.’ (Participant 4)

Not only did the women experience changes in their intimate relationships; some of the women narrated that they separated with their life partners, while some mentioned that the separation also extended to the life partners forming poor bonds with their children, including diminished communication:

‘The relationship was bad he even left and left me with the pregnancy alone.’ (Participant 19)

‘The relationship worsened, and we separated …’ (Participant 4)

‘We separated during pregnancy. I was the reason because I was angry, he infected me.’ (Participant 6)

‘We were distant he never wanted to be close to me anymore.’ (Participant 20)

#### Sub-theme 1.2: The lack of support from family members

In this sub-theme, the participants reported that sharing their HIV status with family members was daunting, affecting their family dynamics and support systems. Furthermore, revealing their status to family members was not well received. The participants reported:

‘They were shocked on why did I keep quiet for so long.’ (Participant 4)

‘My mom started by being angry and as time went by, she became fine.’ (Participant 6)

‘I do not even know what to think because the way we do not get along as family members.’ (Participant 10)

‘But she was a bit disappointed.’ (Participant 18)

‘I had to hide my child road to health booklet, I lacked support.’ (Participant 7)

#### Sub-theme 1.3: Stigmatising behaviours from friends and colleagues

Disclosure of the HIV status of the participants not only affected their relationships with family members, but also extended to friends and colleagues. The participants revealed that they grappled with disclosing to close friends. They began to experience changes in the way they interact with friends and colleagues. Their friends and colleagues presented with stigmatising behaviour towards the women following the disclosure of their HIV status. Participants indicated that:

‘They did not react in the same way … but there were those who will make jokes out of it. Some colleagues would make silly comment … The way the work environment was toxic, a part of me went through depression.’ (Participant 5)

‘I thought people were going to gossip about me.’ (Participant 14)

‘You know the stigma that is being associated with being born infected with HIV and be ridiculed by the community.’ (Participant 18)

Some of the participants did not directly experience these stigmatising behaviours; however, they were fearful that if they disclose to friends and colleagues, they may start experiencing negative reactions from their colleagues, friends, and families. The participants said:

‘I would not be happy if people at my place knows about my condition, they would be pointing fingers at me, gossiping about me.’ (Participant 13)

‘I was asking myself questions like, “will he leave me?” Will he say he took it from me “if he tested negative and I am positive what will he say, will he leave me?” These were the questions bothering me … You know how families are they will think you were sleeping around.’ (Participant 3)

‘I was asking myself questions like, “How will people accept me?” “What will the father of my child say because he was HIV negative?”’ (Participant 16)

### Theme 2: The role of psychosocial support to improve interpersonal relationships

Participants in this study cited the need for psychosocial support to help them improve their interpersonal relationships while coming to terms with being diagnosed with HIV during pregnancy. Participants referred to the support of significant others as being helpful to achieve solid interpersonal relationships while dealing with the hardships of being diagnosed with HIV during pregnancy. This theme is supported by the sub-themes below.

#### Sub-theme 2.1: The value of sound interpersonal relationships with significant others

Some of the participants mentioned that the disclosure of their HIV status to significant others led to more support, which made it easy for them to deal with the diagnosis during pregnancy. Sound interpersonal relationships for women diagnosed with HIV during pregnancy play an important role in providing emotional support, as well as overall life satisfaction and mental health during difficult times. The participants said:

‘With my knowledge finding out I was HIV positive was like any other sickness because I had someone who supported me. I had support and I made peace that this is a sickness I am going to live with.’ (Participant 8)

‘Talk to them nicely and patient so they can understand, some patients cannot understand but try.’ (Participant 17)

‘My family is very supportive, they will not be like you are sick we are not eating with you, or we do not share utilities with you, they do not have discrimination I share with them everything.’ (Participant 9)

‘They guided me that if I drink my treatment well, I am going to be fine.’ (Participant 12)

#### Sub-theme 2.2: Counselling services

The women in this study indicated that they needed counselling to help them process their emotions and build resilience. as dealing with the diagnosis of HIV during pregnancy could be arduous. Counselling can help them navigate the emotional challenges resulting from their experience of altered interpersonal relationships:

‘I think talking to an individual on a one-on-one session would assist but also getting the experience of different individuals would also be beneficial.’ (Participant 5)

‘Counselling everyday mind due [*sic*] some families are not supportive at all; we have seen it with other families out there.’ (Participant 1)

‘I need counselling, I think I might be better.’ (Participant 10)

‘You might want to talk and counsel the person.’ (Participant 2)

Furthermore, one of the participants mentioned that skilled personnel should provide counselling to ensure complete recovery:

‘I feel like there should be someone to ask if they are feeling fine and be offered counselling to bring them back to reality. I wish there can be people who are trained to counsel individuals.’ (Participant 4)

#### Sub-theme 2.3: Support groups

Participants in this study mentioned support groups as an integral part of the support that can be provided to women who are diagnosed with HIV during pregnancy. They said the following:

‘It is important for the clinic staff to give each of them support.’ (Participant 1)

‘They should at least work on a group of 5 to 10 people and give them counselling mind due [*sic*] after receiving the news.’ (Participant 4)

‘You should give them counselling or create some support groups.’ (Participant 5)

‘I would have preferred a group session because other woman would be sharing their experiences also.’ (Participant 6)

The participants further indicated that support groups will allow them to vent their frustrations and learn from the experiences of other women who have been diagnosed with HIV during pregnancy:

‘Group counselling because there are different people who will vent about their stories and what they came across. It will be easy to learn from their experiences and feel much better that you are not alone.’ (Participant 18)

## Discussion

The study findings revealed that being diagnosed with HIV during pregnancy has a negative impact on the interpersonal relationships of women. Sound interpersonal relationships are an essential building block for positive interaction with others. Sound interpersonal relationships bring about gratifying engagements with others and provide pleasure and satisfaction, particularly while enduring difficulties.^28^ In the absence of sound interpersonal relationships, interactions may be chaotic. Being diagnosed with a chronic condition may worsen interpersonal relationships with close individuals. The findings of this study revealed that the diagnosis of HIV during pregnancy for the participants brought instability and further altered their relationships with significant others, negatively impacting on their mental health. The reactions of the loved ones of the participants had a significant impact on their mental and emotional well-being. Therefore, addressing the emotional and social needs of women living with HIV is crucial for their well-being. Similarly, the findings of this study relate to those of people living with mental illness, whose interpersonal relationships were also affected following the diagnosis of mental illness, indicating the difficult experience following disclosing one’s chronic condition.^29^ Likewise, in this study, the life partners also displayed negative attitudes toward women diagnosed with HIV during pregnancy. These findings are similar to a study by Habiba et al.,^30^ where intimate partners revealed that they experience pessimistic responses from their life partners. In other contexts, a minority of older women also endured changes in their relationships, including being blamed by their life partners, resulting in reduced sexual activity in their relationships.^31^ Not only were their reactions negative, but the diagnosis of HIV also led to reduced intimacy and, eventually, separation.

Family members, friends, and colleagues play an important role in terms of providing support to any person during difficult times. However, the disclosure of HIV status to significant others is linked to complexities such as rejection. Participants in a study conducted by Chapman^32^ revealed that they experienced rejection from friends and families in the form of withdrawal from the relationship, and some were cut off from family interactions. Similarly, the findings of this study revealed that support for women following HIV diagnosis was lacking. It became difficult for the participants, as they received emotional responses such as anger, shock, and disappointment from their parents. These findings are no different from other contexts; participants in a study conducted in the Nordic countries indicated that the lack of support from family and friends was experienced as challenging.^33^ Seemingly, the diagnosis of HIV is linked to family disruptions, not only for pregnant women during pregnancy but for any other person in general.^34^

Not only did the participants of this study experience challenges with family members; friends and colleagues also displayed stigmatising behaviours. Some of the friends and colleagues made utterances, including jokes, about their HIV status. The expression of the participants in this study shared similar characteristics with the findings of Brittain et al.,^35^ where young pregnant women who disclosed their HIV status experienced profound HIV-related stigma. One study found that people living with HIV experienced professional difficulties after revealing their HIV status, where in some cases it led to dismissal from work.^34^ As such, disclosing HIV status at the workplace is linked to negative impact on work relations, as reported by the participants in this study.

In this study, it emerged that psychosocial support services could positively impact the recovery journey of women and, in turn, could improve their interpersonal relationships. Therefore, building meaningful relationships that bring a sense of purpose and joy is important for pregnant women diagnosed with HIV. Psychosocial support services, such as advocating for sound interpersonal relationships, counselling services, and support groups, can help women to be more resilient during this time. Reciprocally, while psychosocial support services are effective in improving relationship quality and emotional coping, they could also be helpful in improving the health outcomes of pregnant women diagnosed with HIV during pregnancy.^36^ Counselling serves as a supportive process to help people solve problems and personal conflicts. The participants in this study also mentioned that counselling could be beneficial for them to find healing, as well as to allow them to process the news about their HIV status. Similarly, other studies highlighted the importance of counselling as an essential tool to understand the situation.^37^

Strong social support acts as a buffer to distress and is essential for enhancing physical and mental health.^38^ In addition, support groups are effective for people dealing with similar situations to learn from each other, particularly about coping mechanisms. Support groups facilitate a quicker HIV status acceptance through building advocacy networks, which allows the women to have a safe space to voice their health needs.^39^ The findings of this study highlighted the importance of these support groups to ensure that pregnant women diagnosed during pregnancy can share their experiences with other women in similar situations. They will not only benefit the women, but also the relationships of women who are diagnosed with HIV during pregnancy. Building strong social networks may be helpful to women, especially with regard to prioritising their own well-being and developing emotional capacity to deal with the response of others towards them following the disclosure of their HIV status.

### Strengths and limitations

An adequate number of participants were interviewed until saturation point to ensure that rich thick descriptions were obtained from the data collection. The sensitivity and nature of the study could have introduced social desirability bias, where the participants only reported the information that they thought could be socially acceptable. Additionally, the study focused on pregnant women in a specific context; therefore, applicability to other contexts may not be possible.

### Implications or recommendations

Based on the findings of this study, it is essential to improve psychosocial support services by implementing comprehensive counselling programmes, support groups, and increasing life partner engagement. These are crucial to combat stigma and discrimination in the communities and at the workplace by providing community education on HIV, strengthening workplace policies, and enhancing family support systems. Therefore, efforts must be made to establish workplace assistance and policies that aim to protect HIV-positive employees, including pregnant women. Incorporating mental health services into ANC and providing disclosure support are essential components of holistic care during pregnancy. Furthermore, policy implications include advocating for the rights of HIV-positive pregnant women and developing guidelines for healthcare providers to facilitate constructive interpersonal relationships with these women. Implementing these recommendations could significantly improve health outcomes, mental well-being, and interpersonal relationships for women diagnosed with HIV during pregnancy. Furthermore, these interventions could create a more supportive environment, reducing the negative impact of HIV diagnosis on interpersonal relationships, and overall quality of life for pregnant women diagnosed with HIV during pregnancy.

## Conclusion

The findings of this study revealed that HIV diagnosis during pregnancy has a significant impact on the interpersonal relationships of women. This results in changes in interpersonal relationships with life partners, the lack of support from family, as well as stigmatising behaviour from friends and colleagues and, ultimately, has an impact on the mental health of the women. The findings of this study provided crucial insights on the role of support in the management of women who are diagnosed with HIV during pregnancy. It is important to consider multiple methods of psychosocial support services that need to be incorporated into the treatment of pregnant women diagnosed with HIV, to improve their interpersonal relationships. The findings could be used to inform policymakers and the Department of Health on the need to consider ongoing support strategies during ANC for pregnant women diagnosed with HIV, including their intimate partners, families, friends, and colleagues.
